# Direct and indirect ENSO modulation of winter temperature over the Asian–Pacific–American region

**DOI:** 10.1038/srep36356

**Published:** 2016-11-08

**Authors:** Marco Y. T. Leung, Wen Zhou

**Affiliations:** 1 Guy Carpenter Asia-Pacific Climate Impact Centre, School of Energy and Environment, City University of Hong Kong, Hong Kong SAR, China; 2 City University of Hong Kong Shenzhen Research Institute, Shenzhen, China

## Abstract

In this study, the direct and indirect atmospheric responses over the Asian**–**Pacific–American region to the El Niño**–**Southern Oscillation (ENSO) are documented. Since ENSO is likely to induce the northward displacement of the East Asian trough (NDEAT), some of the influence of ENSO on the Asian**–**Pacific–American region is possibly indirect and acts by inducing NDEAT. To separate corresponding influences of ENSO and NDEAT, partial regression is utilized. It is noted that temperature variations in the East Asian**–**Western Pacific region are controlled mainly by NDEAT. In contrast, ENSO demonstrates a weak direct relation to the temperature variation over the East Asian**–**Western Pacific region. This suggests that the influence of ENSO on this region is indirect, through modulation of NDEAT. On the other hand, temperature variation over the tropical eastern Pacific is dominated by ENSO forcing. Finally, temperature variation over the eastern North American**–**Western Pacific region is controlled by both ENSO and NDEAT. Nevertheless, their influences on temperature and circulation over this region tend to offset each other. This implies that temperature variation is controlled by their relative strengths.

The El Niño**–**Southern Oscillation (ENSO) is a dominant ocean and atmospheric coupling phenomenon over the tropical Pacific^1^,^2^. In the ocean, anomalous warm sea surface temperatures are found in the tropical Eastern Pacific in ENSO. To the west, an anomalous horseshoe-shaped area of cold sea surface temperatures can be observed. In the atmosphere, an eastward shift of the convective center over the tropical Western Pacific is associated with anomalous sea surface temperatures during El Niño winters. This changes both the zonal overturning cell (Walker cell)^3^,^4^ and meridional overturning cell (Hadley cell)^5^. Aside from its modulation on overturning circulation, ENSO also induces a Rossby wave train, originating from the tropical central Pacific and moving to North America^6^,^7^,^8^,^9^. This shows the possible linkage of ENSO with circulation changes in remote regions.

Because ENSO is linked to circulation variability in remote regions, previous studies have investigated surface temperature and wind responses to ENSO in different regions^10^,^11^. In El Niño winters, the Rossby wave train, excited in the tropics, propagates to higher latitudes and leads to a deeper and eastward-shifted Aleutian low^1^,^11^. In association with this change in the Aleutian low, warmer and moister air is advected to the western coast of North America. The wave train, on the other hand, results in a colder and wetter southwestern North America. El Niño also contributes to the variability of the East Asian winter monsoon^12^,^13^,^14^ by inducing anomalous anticyclonic flow in the lower troposphere over the western North Pacific^15^. Therefore, ENSO results in climate variation in remote regions by changing the large-scale circulation.

Even with the exclusion of ENSO forcing, the East Asian winter monsoon is linked to the downstream North American winter monsoon in association with internal atmospheric dynamic processes^8^,^9^,^16^,^17^,^18^. Hence, the climate variation over the Asian**–**Pacific–American region is also possibly caused by changes in internal atmospheric dynamic processes. However, previous studies found that ENSO affects these processes by modulating atmospheric climate systems^4^,^16^,^19^,^20^,^21^,^22^. One of these systems is a stationary trough in the midtroposphere over East Asia, termed the East Asian trough (EAT)^23^,^24^,^25^,^26^. Recent studies found the north-south displacement of the EAT to be closely related to temperature variation in the southern part of East Asia^4^,^14^,^27^. It has also been found that the northward displacement of the EAT (NDEAT) is possibly initiated by ENSO^4^. This illustrates the potential indirect forcing of ENSO on the East Asian and North American winter monsoons, through changing the internal atmospheric dynamic processes. Therefore, the Asian**–**Pacific–American region, subjected to the direct or indirect forcing of ENSO, is not well distinguished or classified. This study is carried out to examine these two types of ENSO forcing on climate variability over the Asian**–**Pacific–American region at both seasonal and synoptic time scales.

## Results

**Figure 1:**
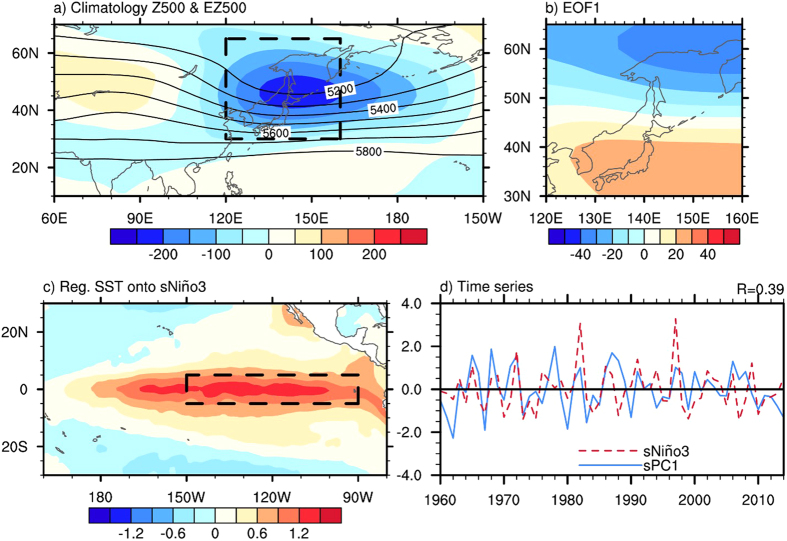
Spatial and temporal variations of NDEAT and ENSO. (**a**) Climatological height (contours) and eddy height (shading) at 500 hPa. The dashed box in (**a**) shows the selected region for the EOF analysis. (**b**) First EOF pattern. (**c**) Regression of sea surface temperature onto sNiño3. The dashed box in (**c**) indicates the region for the computation of sNiño3. (**d**) Time series of NDEAT (sPC1; solid blue line) and ENSO (sNiño3; dashed red line). This figure is generated with the NCAR Command Language (Version 6.2.1) [Software]. (2014). Boulder, Colorado: UCAR/NCAR/CISL/TDD. http://dx.doi.org/10.5065/D6WD3XH5.

To measure and investigate ENSO and NDEAT, different parameters are used (see methods). For ENSO, the Niño3 index is utilized. This index is the average of sea surface temperatures over the region (5°N to 5°S and 90°W to 150°W; Fig. 1c). For NDEAT, we perform empirical orthogonal function (EOF) analysis to a geopotential height of 500 hPa over the region (30° to 65°N and 120° to 160°E; Fig. 1a)^27^. The first EOF represents the northward displacements of the EAT (Fig. 1b), respectively. Hence, the time series of the first EOF (PC1) is used as an index of NDEAT. The standardized values of Niño3 (sNiño3) and PC1 (sPC1) are illustrated in Fig. 1d. The correlation between the two indexes is 0.39 and passes the 0.05 significance test. This implies that NDEAT is likely to occur during El Niño winters. To delineate the impacts on different regions at seasonal and synoptic scales, we investigate the two time scales separately.

### Seasonal variation

**Figure 2:**
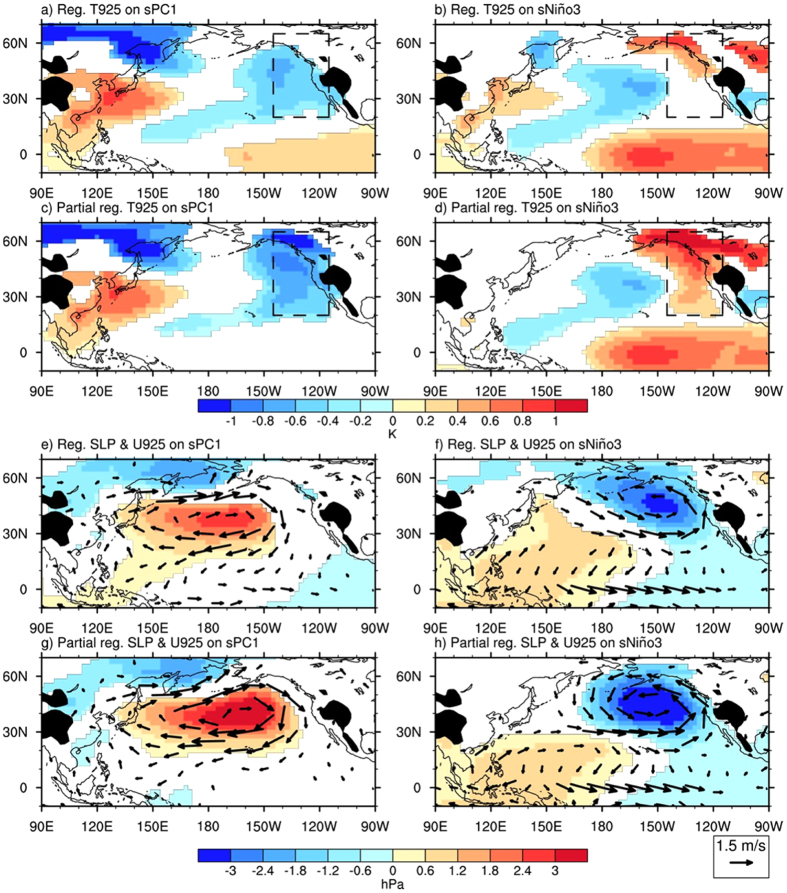
Seasonal responses of temperature and circulation to NDEAT and ENSO. Regressions of temperature at 925 hPa (T925) onto sPC1 (**a**) and sNiño3 (**b**). (**c**,**d**) Are similar to (**a**,**b**), but for partial regressions. The dashed boxes represent the regions of western North America and the midlatitude Eastern Pacific. (**e**–**h**) Are similar to (**a**–**d**), but for sea level pressure (SLP; shading) and horizontal wind at 925 hPa (U925; vectors). Only values over the 5% significance level are depicted. This figure is generated with the NCAR Command Language (Version 6.2.1) [Software]. (2014). Boulder, Colorado: UCAR/NCAR/CISL/TDD. http://dx.doi.org/10.5065/D6WD3XH5.

Based on statistical analysis, NDEAT is found to be significantly related to temperature variation over the Asian**–**Pacific–American region. Warmer-than-normal temperatures are found in the lower troposphere over the mid- to low-latitude East Asian–Western Pacific region and the tropical Eastern Pacific (Fig. 2a). Colder-than-normal temperatures are found over the high-latitude East Asian–Western Pacific region and the midlatitude Western American–Eastern Pacific region. A similar spatial structure can be observed in the temperature responses to ENSO, as shown in Fig. 2b. Nonetheless, the signals of temperature responses to ENSO are remarkably weaker over the East Asian–Western Pacific region and stronger over the tropical Eastern Pacific. In addition, a reversed polarity of temperature response is noted over the coastal regions of western North America for the two climate variations. Accordingly, the temperature variation over most of the Asian**–**Pacific–American region except the coastal regions of western North America in conjunction with ENSO and NDEAT is similar.

As mentioned above, ENSO is also a possible cause of NDEAT. The significant temperature variations in Fig. 2b are possibly contributed by indirect ENSO forcing and through inducing NDEAT. To separate the direct and indirect ENSO forcing on different regions, partial regressions are employed. By fixing the ENSO effect, the signals over the East Asian–Western Pacific region remain significant to NDEAT (Fig. 2c). The negative signal over the midlatitude Western American–Eastern Pacific region intensifies and extends northward. However, the positive signal over the tropical Eastern Pacific is suppressed. By fixing the NDEAT effect, most of the signals over the East Asian–Western Pacific region become insignificant to ENSO (Fig. 2d). The positive signal over the midlatitude Western American–Eastern Pacific region intensifies and extends southward. Consequently, the temperature over the East Asian–Western Pacific region is indirectly controlled by ENSO and through modulating NDEAT. The temperatures over the tropical Eastern Pacific are directly controlled by ENSO. Two types of ENSO forcing demonstrate opposite effects over the midlatitude Western American–Eastern Pacific region.

In order to explain the temperature responses of different regions to the indirect ENSO forcing, regressions and partial regressions of horizontal wind at 925 hPa and sea level pressure onto sPC1 are illustrated in Fig. 2e,g. With indirect ENSO forcing, a negative center of SLP can be found in northeast Asia and a positive center can be found in the midlatitude Central Pacific concurrent with NDEAT. This depicts a northward displacement of the Aleutian low and a weakened Siberian high, by which northerly wind over East Asia is weakened. Hence, the associated southward transport of cold air is also suppressed, which leads to a warmer-than-normal mid- to low-latitude East Asian–Western Pacific region. It is worth noting that the anticyclonic flow over the midlatitude Central Pacific transports cold air from the north to the midlatitude Western American–Eastern Pacific region and results in the colder-than-normal temperatures (Fig. 2a,c).

In addition, regressions and partial regressions of U925 and SLP onto the standardized Niño3 are shown in Fig. 2f,h. Anomalous positive SLP and anticyclonic flow are found over the tropical Western Pacific. The direct forcing of ENSO on the wind and southward transport of cold air over East Asia is weak (Fig. 2d). In the midlatitude Central to Eastern Pacific, there is a center of anomalous negative SLP and cyclonic wind. The cyclonic flow carries warm air from the south to the midlatitude Western American–Eastern Pacific region and leads to the warmer-than-normal temperatures (Fig. 2b,d).

### Synoptic variations

Aside from the seasonal responses, we also examine synoptic responses to the direct and indirect ENSO forcing. To measure the changes in weather-scale temperature variance and their relation to seasonal responses, synoptic temperature variation (2 to 9 days) and the Eady growth rate are used (see methods).

**Figure 3:**
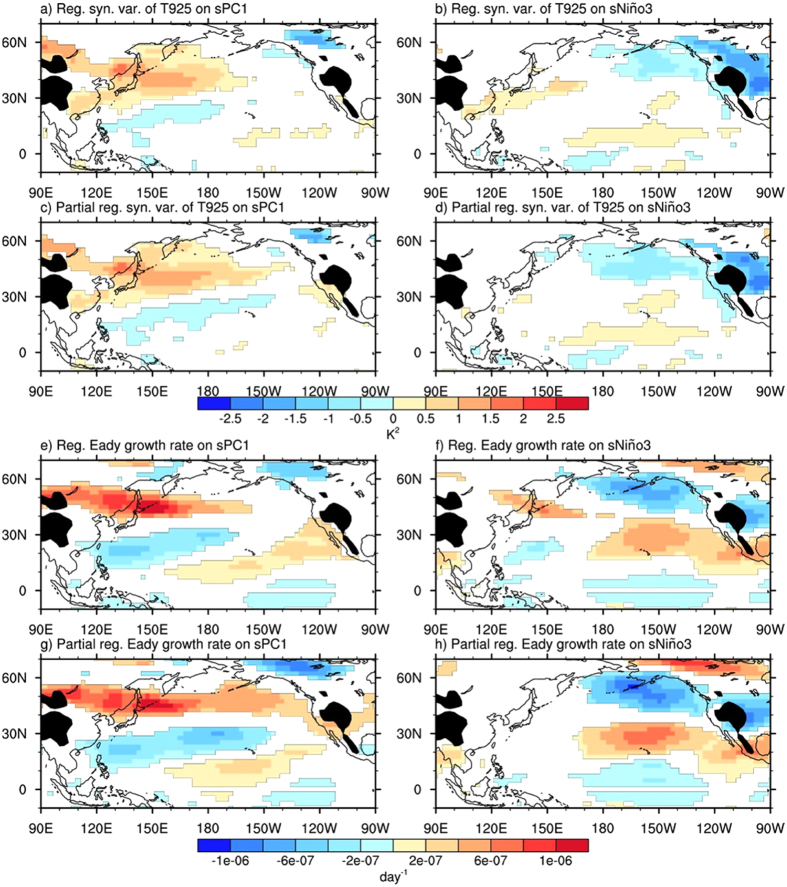
Changes in synoptic temperature variation and eddy development in response to NDEAT and ENSO. Regressions of synoptic temperature variation at 925 hPa onto sPC1 (**a**) and sNiño3 (**b**). (**c**,**d**) Are similar to (**a**,**b**), but for partial regressions. (**e**–**h**) Are similar to (**a**–**d**), but for Eady growth rate. Only values over the 5% significance level are depicted. Regions above 1500 m are masked. This figure is generated with the NCAR Command Language (Version 6.2.1) [Software] (2014). Boulder, Colorado: UCAR/NCAR/CISL/TDD. http://dx.doi.org/10.5065/D6WD3XH5.

Changes in synoptic variation can be found in the Asian**–**Pacific–American region concurrent with the two types of ENSO forcing. Regressions and partial regressions of synoptic temperature variance at 925 hPa to sPC1 and sNiño3 are illustrated in Fig. 3a–d. With NDEAT, the temperature variances over the East Asian–Western Pacific region increase (Fig. 3a,c). It should also be noted that NDEAT is also responsible for the decrease in synoptic variance in central Canada and the increase in the western Rocky Mountains. However, most of the midlatitude Western American–Eastern Pacific region is controlled by ENSO, over which the synoptic temperature variance is strongly suppressed (Fig. 3b,d). On the other hand, direct ENSO forcing is weak over the East Asian–Western Pacific region. Accordingly, direct and indirect ENSO forcing are related to changes in synoptic temperature variance over the Asian**–**Pacific–American region.

As synoptic temperature variation is a result of the passage of extratropical eddies, changes in the development of these eddies are also studied. Regressions and partial regressions of the Eady growth rate onto sPC1 and sNiño3 are presented in Fig. 3e–h. With indirect ENSO forcing, an increase in the Eady growth rate is noted over the midlatitude East Asian–Western Pacific region, along with NDEAT (Fig. 3g). This shows that the intensification of extratropical eddies is enhanced. With direct ENSO forcing, the decrease in the Eady growth rate over the midlatitude Western American–Eastern Pacific region implies a suppression of intensification of extratropical eddies (Fig. 3h). The changes in intensification of extratropical eddies are indirectly related to the changes in the seasonal meridional temperature gradient (Fig. 2c,d).

## Discussion

**Figure 4:**
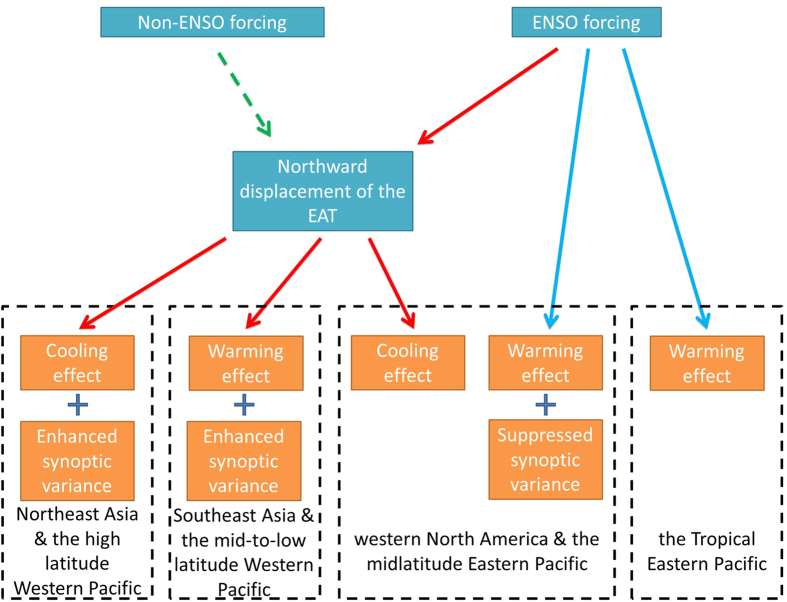
Schematic diagram of the direct (blue arrows) and indirect (red arrows) ENSO forcing on different parts of the Asian–Pacific–American region. The modulation of Non-ENSO forcing is indicated with the green dashed arrow.

In this study, the direct and indirect ENSO forcing on both seasonal and synoptic temperature responses over the Asian**–**Pacific–American region are documented. The direct and indirect ENSO forcing are identified via partial regression. The findings are briefly summarized in a schematic diagram (Fig. 4). ENSO exerts direct forcing on the seasonal temperature variation over the tropical Eastern Pacific. There is also an indirect ENSO forcing on the temperature variation over the East Asian–Western Pacific region, through inducing NDEAT. These temperature variations are contributed mainly by anomalous wind in the lower troposphere (Fig. 2g,h). The direct and indirect ENSO forcing show opposite impacts on horizontal wind and temperature in the lower troposphere over the midlatitude Western American–Eastern Pacific region. Hence, the temperature variation is controlled by two types of forcing and depends on their relative strengths. To demonstrate the control of their relative strengths on the temperature variation, we also calculated the temperature over the region (20° to 65°N and 115 to 145°W; dashed box in Fig. 2) and found correlations of 0.43 with sPC1 and −0.28 with sNiño3. This shows a remarkably higher correlation (0.61) with the difference in the two indexes (sPC1- sNiño3).

Apart from the controlling forcing on seasonal temperature variation, the direct and indirect ENSO forcing also modulate synoptic variance over the Asian**–**Pacific–American region. With indirect ENSO forcing, changes in the seasonal circulation enhance the intensification of extratropical cyclones over the East Asian–Western Pacific region in association with ENSO-induced NDEAT. This increases synoptic temperature variance. With direct El Niño forcing, extratropical cyclones over the Western American–Eastern Pacific region are suppressed, by which synoptic temperature variance is decreased. Changes in the intensification of extratropical cyclones are concomitant with the changes in meridional temperature gradient that occur with the two types of ENSO forcing.

As mentioned above, the correlation between ENSO and NDEAT passes the 0.05 significance test. However, it cannot explain the total variance of NDEAT. We removed the geopotential height responses to ENSO before the computation of EOF. Nonetheless, the first EOF pattern after the removal of the response to ENSO demonstrates high similarity to that before the removal (figure not shown). Therefore, NDEAT can be also initiated by unknown processes called Non-ENSO forcing (Fig. 4). Since Non-ENSO forcing possibly supports or counteracts the ENSO forcing on NDEAT, it results in the magnitude and phase variations of NDEAT. This suggests that Non-ENSO forcing is important to the relative strength of ENSO and NDEAT. Hence, further study is needed to identify this unknown forcing.

## Methods

### Data

To investigate the atmospheric responses to NDEAT and ENSO, different parameters including geopotential height (Z), horizontal wind (U), sea level pressure (SLP), and air temperature (T) from the National Centers for Environmental Prediction‒National Center for Atmospheric Research (NCEP‒NCAR) are employed in this study^28^. Additionally, a sea surface temperature (SST) data set from the Met Office Hadley Centre is utilized for measuring the variation of ENSO^29^.

### Synoptic temperature variance

To extract the synoptic signals (2 to 9 days) from temperature, we employed the Lanczos filter as a band pass filter. The weight of the filter is 9 days. Therefore, the synoptic temperature variance is computed by the monthly mean of the squared band-pass-filtered daily temperature.

### Eady growth rate

The Eady growth rate is widely used to measure the intensification of extratropical cyclones^30^,^31^. The equation of the rate is 
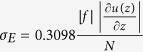
 where 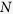 is the Brunt–Väisälä frequency 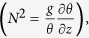 *g* is gravitational acceleration, *θ* is potential temperature, *f* is the Coriolis parameter, and *u*(*z*) is the vertical profile of the eastward wind component.

## Additional Information

**How to cite this article**: Leung, M. Y. T. and Zhou, W. Direct and indirect ENSO modulation of winter temperature over the Asian–Pacific–American region. *Sci. Rep.* **6**, 36356; doi: 10.1038/srep36356 (2016).

**Publisher’s note:** Springer Nature remains neutral with regard to jurisdictional claims in published maps and institutional affiliations.
